# Cannabis Use, Schizotypy and Kamin Blocking Performance

**DOI:** 10.3389/fpsyt.2021.633476

**Published:** 2021-11-23

**Authors:** Christopher Dawes, Andrea Bickerdike, Cian O'Neill, Sarah Carneiro Pereira, John L. Waddington, Paula M. Moran, Colm M. P. O'Tuathaigh

**Affiliations:** ^1^School of Psychology, University Park, University of Nottingham, Nottingham, United Kingdom; ^2^Department of Sport, Leisure, and Childhood Studies, Munster Technological University, Cork, Ireland; ^3^Psychological Sciences Research Institute, Université Catholique de Louvain, Louvain-la-Neuve, Belgium; ^4^School of Pharmacy and Biomolecular Sciences, Royal College of Surgeons in Ireland, Dublin, Ireland; ^5^Medical Education Unit, School of Medicine, University College Cork, Cork, Ireland

**Keywords:** cannabis, schizotypy, Kamin blocking effect, aberrant salience, psychosis

## Abstract

Cannabis use has been associated with increased risk for a first episode of psychosis and inappropriate assignment of salience to extraneous stimuli has been proposed as a mechanism underlying this association. Psychosis-prone (especially schizotypal) personality traits are associated with deficits in associative learning tasks that measure salience allocation. The aim of this study was to examine the relationship between history of cannabis use and Kamin blocking (KB), a form of selective associative learning, in a non-clinical sample. Additionally, KB was examined in relation to self-reported schizotypy and aberrant salience scale profiles. A cross-sectional study was conducted in 307 healthy participants with no previous psychiatric or neurological history. Participants were recruited and tested using the Testable Minds behavioural testing platform. KB was calculated using Oades' “mouse in the house task”, performance of which is disrupted in schizophrenia patients. Schizotypy was measured using the Schizotypal Personality Questionnaire (SPQ), and the Aberrant Salience Inventory (ASI) was used to assess self-reported unusual or inappropriate salience. The modified Cannabis Experience Questionnaire (CEQm) was used to collect detailed history of use of cannabis and other recreational drugs. Regression models and Bayesian *t*-tests or ANOVA (or non-parametric equivalents) examined differences in KB based on lifetime or current cannabis use (frequent use during previous year), as well as frequency of use among those who had previously used cannabis. Neither lifetime nor current cannabis use was associated with any significant change in total or trial-specific KB scores. Current cannabis use was associated with higher Disorganised SPQ dimension scores and higher total and sub-scale values for the ASI. A modest positive association was observed between total KB score and Disorganised SPQ dimension scores, but no relationships were found between KB and other SPQ measures. Higher scores on “Senses Sharpening” ASI sub-scale predicted decreased KB score only in participants who have not engaged in recent cannabis use. These results are discussed in the context of our understanding of the effects of long-term cannabis exposure on salience attribution, as well as inconsistencies in the literature with respect to both the relationship between KB and schizotypy and the measurement of KB associative learning phenomena.

## Introduction

Increased attention to irrelevant stimuli is postulated to represent a core disturbance central to the signs and symptoms of psychosis (1–4). Inappropriate attribution of significance to neutral stimuli has been linked with dysregulation of striatal dopaminergic transmission (4–7) and is observed in both patients with a psychotic disorder (8–10). Similar deficits have been reported in individuals high in psychometrically identified schizotypy, a multidimensional construct with positive, negative, and disorganised symptom dimensions consistent with those described in schizophrenia (11–13). This has led to the suggestion that disturbance in salience allocation processes may mediate the link between neurobiological and psychological risk factors and emergence of psychotic symptoms (14, 15). Aberrant salience has also been postulated to be central to our understanding of psychosis risk and mechanisms that contribute to the development of psychotic symptoms (3, 6).

Cannabis use has been linked with increased risk for a first episode of psychosis (16–18). Acute cannabis use can produce transient psychotic symptoms (18–20) and may precipitate a psychotic episode in individuals with a pre-existing psychotic disorder (21, 22). Cannabis-induced modulation of dopaminergic function has been linked with increased vulnerability for psychosis following long-term cannabis exposure (23) and, consistent with the continuum model of schizophrenia, several studies have shown a relationship between long-term cannabis use and schizotypal traits in the general population (24–29). Schizotypal traits have also been linked with increased frequency of reported psychosis experiences in cannabis users (29, 30). Studies examining cognitive function in cannabis users have demonstrated deficits in measures of attentional processing that require selection of relevant from irrelevant content (23, 31–33). Similarly, cannabis use is associated with higher scores on the Aberrant Salience Inventory (ASI), a questionnaire designed to measure tendency towards assignment of salience to inconsequential stimuli (15, 34). Disturbance in salience processing may, at least in part, mediate the link between frequency of cannabis use and preponderance of schizotypal traits (15).

Assignment of salience can be measured experimentally in learning tasks where allocation of associability to a stimulus is rendered more or less correctly depending on reinforcement history. A selection of such associative learning tasks has been investigated in psychosis, most prominently Kamin (or conditioned) blocking (KB) and latent inhibition (35–37). In the KB paradigm, prior learning to one stimulus, CS1, decreases subsequent learning to an added stimulus, CS2, when both stimuli are later presented in a reinforced compound, CS1-2 (38). In essence, learning of the association between CS2 and the reinforcing (unconditioned) stimulus (US) is decreased because it has been blocked by the prior association between CS1 and the US. It has been suggested that this blocking effect may reflect a failure to shift attention or associability from the erstwhile highly productive CS1 to the novel CS1-CS2 compound stimulus. Human and rodent studies have implicated the striatum, amygdala and prefrontal cortex as critical structures involved in blocking performance (39–41). In a recent study, both pharmacological disruption of inhibition in the ventral tegmental area (VTA) and inactivation of the nucleus accumbens (NAc) during compound stimulus learning reduced KB, consistent with the role of dopamine in prediction error signalling and reinforcement learning more generally (41). We have previously demonstrated modulation of KB in animals following pharmacologically-induced enhancement of dopaminergic activity (42, 43).

Disruption of KB, as indicated by greater than expected learning accruing to CS2, has been observed in patients with schizophrenia (36, 37, 44–47). While initial studies indicated that KB deficits were associated with severity of positive symptoms (44), subsequent studies have largely linked such deficits with a negative symptom profile in patients with schizophrenia (27, 36, 47). Evidence for a link between schizotypal traits in health individuals and variation in KB is more uncertain. Moran et al. (36) demonstrated that KB disruption, specifically during earlier trials, was associated with increased schizotypy scores (particularly the cognitive disorganisation and unusual experiences sub-scales of the O-LIFE scale), in a manner that was observed in both non-paranoid patients with schizophrenia and healthy controls. Subsequent studies have either shown a relationship between blocking and the negative dimension of schizotypy (48), or have failed to show any relationship with psychometrically-defined schizotypy (49). No studies to date have investigated any relationship between salience allocation measured as KB, cannabis use, and schizotypy.

The present study examined the KB paradigm in cannabis users relative to cannabis non-users. Consistent with reported effects of cannabis on attention and associative learning and on neurotransmitter systems underling these functions, we hypothesised that deficits in KB would be more evident in individuals who use cannabis more frequently. This study also assessed the putative relationships between KB, psychometrically-defined schizotypy and self-reported aberrant salience. We predicted that individuals with a negative schizotypy profile and/or reporting high aberrant salience would exhibit reduced KB in a similar direction to that reported in patients with schizophrenia.

## Methods

### Design and Participants

This cross-sectional study was conducted using the Testable Minds (www.testable.org) platform, a subject pool for psychology experiments with a participant verification system (50). Testing and recruitment platforms such as that used in the present study can provide researchers access to hitherto less accessible study populations such as non-treatment seeking cannabis users (51). Testable Minds employs verification checksat sign up and for each study participation to minimise the issues (e.g., multiple accounts, “bot” responses) encountered by other such participant pool platforms (52). Participation in this study was restricted to “verified minds”; to qualify as such, participants are required to submit an official photo ID and take a live webcam photo of their face. The photo ID is used to manually verify the first and last names, date of birth, sex, and country of residence. It was reported that, as of June 2020, ~15,000 members are registered in the Testable Minds participant pool, mean age 34 years (SD = 11.5) and 51.7% male. In terms of location, 42% are from USA, 19% from UK, 18% from EU countries, 2% from Canada and 2% from Australia and New Zealand (50).

Testable Minds members were invited to participate in a study with both questionnaire and PC-based task elements. They were informed that the study was designed to examine the relationship between personality and lifestyle factors and attentional function. The inclusion criteria for the study were (i) individuals aged between 18 and 55 years old and (ii) from a predominantly English-speaking location. Participants were excluded if they (i) had a history of neurological disease or brain injury and/or (ii) had been formally diagnosed with a psychiatric illness. Ethical approval was obtained from the Social Research Ethics Committee of University College Cork. All participants provided informed consent and were compensated £5 for their time.

### Questionnaire Measures

Participants were first asked to complete a brief demographics survey, which included questions about age, sex, nationality, and education level. They were then directed to complete the following: Cannabis Experience Questionnaire modified version [CEQmv; (16)]; Schizotypal Personality Questionnaire [SPQ; (53)]; ASI (54).

The CEQmv was administered to collect information on history of cannabis use (including information such as age at first use, duration of use, frequency of use, type), as well as other recreational drugs including tobacco and alcohol (16). In the present analysis, measures of interest were: lifetime cannabis consumption (“ever vs. never”); current cannabis consumption (defined as frequent use of cannabis consumption during the previous 12 months; “current” vs. “other” comparison); frequency of use which referred to the most recent period of cannabis consumption (a six-level categorical response item: never, only once or twice, a few times each year, a few times each month, more than once a week, every day) (15).

Aberrant salience status was measured using the 29-item ASI instrument (54). This self-report measure aims to assess subjective experiences of aberrant salience attribution (1) and yields a score of 0–29; a higher score corresponds to a greater degree of aberrant salience, derived from yes/no responses to 29 statements. The ASI provides a total score calculated as number of positive responses and has the following five subscales: Increased significance–reflecting heightened attribution of salience to stimuli (e.g., item 10: “Do you ever feel the need to make sense of seemingly random situations or occurrences?”); Senses sharpening–this is also consistent with aberrant salience (e.g., item 17: “Has your sense of taste ever seemed more acute?”); Impending understanding–referring to an individual's feeling of perceived significance associated with a psychotic episode (e.g., item 6: “Do you sometimes feel like it is important for you to figure something out, but you're not sure what it is?”); Heightened emotionality–signifying greater anxiety during psychosis, particularly in the early phases of a psychotic episode when the individual attempts to understand the increased importance of stimuli (e.g., item 8: “Do you ever have difficulty telling if you are thrilled, frightened, pained, or anxious?”); Heightened cognition–reflective of the experience of being part of something not apparent to others (e.g., item 25: “Do you ever perceive an overwhelming significance to things that are usually not significant to you?”).

The SPQ is a 72-item index of schizotypy, based on DSM-IIIR criteria for schizotypal personality disorder, that can be measured in the general population. The SPQ comprises nine subscales that form three dimensions: (a) Cognitive-perceptual (including the four subscales “ideas of reference”, “magical thinking”, “unusual perceptual experiences” and “paranoid ideation”). An example of this subscale is the item “Have you often mistaken objects or shadows for people, or noises for voices?”; (b) Interpersonal (related to negative symptoms, including the four subscales “social anxiety”, “no close friends”, “constricted affect” and “paranoid ideation”). An example is the item “I have little interest in getting to know other people”; and (c) Disorganised (including the two subscales “eccentric behaviour” and “odd speech”). An example is item “I sometimes forget what I am trying to say” (55). It has been suggested that conceptual and structural differences exist between the various schizotypy scales (e.g., O-LIFE, SPQ) with respect to the measurement of the disorganisation dimension (56). The SPQ uses a dichotomous response format. A total SPQ score is based on the sum of all nine subscale scores. This scale has high reliability and validity (53).

### Kamin Blocking Task

The present blocking procedure was based on Oades' KB task as described by Moran et al. (36, 37, 40), and minimally adapted for delivery via the Testable interface (https://www.testable.org). Participants are instructed that there is a hungry mouse trying to find his cheese in a “house”. On a typical trial, one of six sets of tri- or bi- coloured rectangular bars appears on the screen for 1 s. There are six sets of colours [i.e., set 1 = grey (colour 1), green (colour 2); set 2 = pink (colour 1), brown (colour 2); set 3 = yellow (colour 1), turquoise (colour 2), orange (colour 3); set 4 = red (colour 1), green (colour 2), blue (colour 3); set 5 = purple (colour 1), blue (colour 2), red (colour 3) and set 6 = yellow (colour 1), green (colour 2), pink (colour 3)]. Each set of colours corresponds to a particular location in the “house” which are numbered 1–8. Participants are instructed that (1) there are two main rooms in the house, each with four possible hiding places (1–4 and 5–8) and (2) the mouse will always be in the opposite room for the one where the cheese is hidden. Participants identify the location of the cheese by pressing the corresponding number keys 1–8 on a QWERTY keyboard. If the answer is correct, a piece of cheese appears on the location chosen by the participant and “Correct” appears in the middle of the house plan. If an incorrect choice is made, “Incorrect” is displayed in the middle of the screen. If there is a failure to respond within the response interval period then “No response detected” is displayed.

At the beginning of each fixed length 6-s trial, mouse and colours were superimposed on the house template (the template remained on the screen throughout the experiment, except for breaks between sessions). Moreover, a timer is displayed at the top right of the screen to let participants know how much time they have to respond and to engage them to answer as quickly as possible. At a time of 1 s into the trial, the colours disappear. Subjects were instructed to press one out of eight keyboard keys (four on each hand) as quickly and accurately as possible in a 5-s window to answer. Participants needed to position their left hand in order to have the little finger correspond to number 1 running through to number 4 for the index finger and position their right hand in order to have the index finger correspond to number 5 running through to number 8 for the little finger. Once a key press was detected, feedback information was superimposed on the house template for 2-s and then the next trial followed immediately.

The KB task is run in two conceptual phases and they both take approximately 10 min to complete: (1) Overshadowing (OS) with a test phase and (2) Blocking (BL) with a test phase. In OS there were 2 training phases: (1) 44 trials of bi-coloured rectangular bars were presented (one half of each set 1 and 2) and then (2) 36 trials of tri-coloured bars were shown (one half of each set 3 and 4). Test trials involved 12 trials of colour 1 and 12 trials of colour 3 that are a segment of the tri-coloured sets. Test trials probed how much learning had accrued to individual elements of the tri-colour blocks. In BL there were also two training phases including 44 trials with a bi-colour bar (one half of each set 5 and 6) followed by 36 trial where an additional colour (colour 3) was added to create a tri-colour bar. These sessions were followed by 24 test trials probing how much learning had accrued to individual elements (colours 1 and 3) of the tricolour blocks. As training with the two colour bar fully predicts the spatial location, blocking should be demonstrated as slower or no learning about the third added colour.

A KB score was calculated from the mean difference in latency to respond to the first and third colour bars in the two conditions OS and BL: reaction times (RT) are calculated in milliseconds (ms). Calculation of KB as a function of an overshadowing condition removes any potential confounding influence of overshadowing or stimulus presentation on reaction times. This blocking score was calculated for each of the pairs of test trials (“Trial 1”–“Trial 12”) and the latency difference was averaged across pairs of trials (“KB score”).

### Data Analysis

Normality of data was examined using Shapiro-Wilk tests. As total and individual KB trial scores were normally distributed (after data cleaning), independent sample *t*-tests were used to examine the effects of current or lifetime cannabis use (dichotomous variables in both cases) on total KB score. A one-way analysis of variance (ANOVA) subsequently examined the effect of frequency of cannabis use on total KB score. For individual KB trial scores, a linear mixed-effects model was used with current cannabis use as a fixed effect. Mann–Whitney *U*-tests were used to assess differences in SPQ and ASI scores based on current cannabis use (as these scales were all non-normal and positively skewed, each *p* < 0.001). Linear regression analyses were used to examine whether SPQ and ASI (total and sub-scale scores) predicted total KB score. Finally, correlation analyses were performed between SPQ, ASI, and individual KB trial data using Kendall's rank correlation.

A parallel analysis strategy was applied to the data, in which both frequentist and Bayesian methods were used. This approach was chosen due to some critical null findings of the data, wherein Bayesian methods can discern whether a *p* > 0.05 is the result of a true null effect or insensitivity of the data to detect an effect. For interpreting Bayes Factors, they are the ratio evidence for the alternative hypothesis relative to the null hypothesis (BF_10_). For example, a BF_10_ of 5 means there is five times more evidence for the alternate hypothesis relative to the null. This can be converted into evidence for the null by dividing 1 by the BF_10_ (now the BF_01_). Common cut-off criteria for BFs are as follows: values between 3 and 0.333 can be considered to indicate lack of sensitivity to detect effects (requiring more data), a BF 3 > | <0.333 represents moderate evidence for the alternate and null, respectively, 10 > | <0.1 strong evidence, 30 > | <0.033 very strong evidence, and 100 > | <0.01 decisive evidence (57).

All data were collated and transferred into R Studio (R Studio Team, 2015), which was used to complete the linear mixed-effects models using the lmertest (58) and emmeans (59) packages. Graphs and figures were created in R Studio using ggplot2 (60) and plotly (61). All Bayesian Analyses were conducted in JASP (JASP Team, 2020; https://jasp-stats.org/).

## Results

### Data Cleaning and Study Demographics

Of the 310 participants that attempted the experiment, three were removed due to not completing the KB task. Data cleaning procedures were applied to the remaining 307 participants across total KB score and the 12 individual KB trials. Initially, 230 data points were removed resulting from participants responding slower than 3 s, which were assumed to be lapses in attention. Seven of the 13 KB variables were non-normally distributed according to Shapiro-Wilk tests [all *p* < 0.02 with False Discovery Rate (FDR) correction]. Outliers were removed according to the median +/– 2.5 (the Median Absolute Deviation), resulting in 53 data points being removed across the 13 KB variables (0.013 data points per variable). In total, 7.091% of the data (283 data points) for KB trials were removed. After outlier removal, all variables were normally distributed (all *p* > 0.152, FDR corrected). Linear mixed effects models were used for KB trial scores, which are appropriate for repeated measures data with incomplete data.

Table 1 presents the sociodemographic and cannabis use characteristics of the study sample. Further drug use patterns reported in the present sample are presented in Supplementary Table 1.

**Table 1:** Characteristics of the sample population (*n* = 307).

**Characteristic**	***n***	***%***
**Sex**		
Female	157	(51.1%)
Male	147	(47.9%)
Not specified	3	(1.0%)
**Nationality**		
Irish/British	223	(72.5%)
North American	71	(24.8%)
Other/Not specified	8	(2.6%)
**Highest level of education**		
Secondary level	69	(22.7%)
Post-secondary level	60	(19.7%)
Primary degree	129	(42.4%)
Masters/Doctoral degree	46	(15.1%)
Other/Not specified	3	(1.0%)
**Family history of mental illness**		
Yes	84	(27.4%)
No	172	(56.0%)
Not specified	51	(16.6%)
**Lifetime cannabis use**		
Yes	180	(58.6%)
No	126	(41.0%)
Not specified	1	(0.3%)
**Current cannabis use**		
Yes	75	(24.4%)
No	232	(75.6%)
**Age at first cannabis use**		
Mean age (SD)	18.0	(4.5)
Range	11–40	
**Frequency of cannabis use**		
Every day	28	(9.1%)
Greater than once a week	29	(9.4%)
A few times each month	35	(11.4%)
A few times each year	36	(13.5%)
Only once or twice	60	(19.5%)
Never	118	(38.4%)
Not specified	1	(0.3%)

*Figures presented are number (%) unless stated otherwise*.

Male participants were more likely to have “ever” used cannabis (χ2 = 10.72, *p* = 0.03), but no sex differences were observed for current cannabis use (χ2 = 0.97, *p* > 0.05) or cannabis use frequency (χ2 = 16.43, *p* > 0.05). Family history of mental illness was associated with increased likelihood of current cannabis use (χ2 = 17.54, *p* < 0.001), but no association was observed for lifetime cannabis use (χ2 = 5.23, *p* > 0.05) or frequency of use (χ2 = 16.53, *p* > 0.05). Educational attainment level was not associated with any cannabis use measure (all *p* > 0.05).

Before investigating predictors of KB performance, we first assessed whether KB was exhibited in the sample overall. To do so, the average RT of the 12 individual KB trials were compared against a reference value of 0 ms (i.e., no difference between overshadowing and blocking trials). Multiple one sample *t*-tests (with FDR correction) suggested that Trial 2 (*p* = 0.004), Trial 5 (*p* = 0.004), Trial 7 (*p* = 0.018), and Trial 9 (*p* = 0.004) all significantly differed from 0. The remaining trials did not significantly differ from 0 ms (all *p* > 0.11).

### Replication Analyses

Analyses were conducted to replicate our previous findings between cannabis use and both schizotypy and aberrant salience (15) and to inform upcoming analyses of potential moderating variables. The results of parallel analysis Mann–Whitney *U* tests found that those who currently use cannabis had significantly higher levels of disorganised schizotypy (interpolated median = 6.000) compared to those who do not currently use cannabis (interpolated median = 3.967) at a small effect size (*p* = 0.01, *r*_*rank*−*biserial*_ = 0.196 [0.048, 0.335]). However, the BF reported only anecdotal evidence for the null hypothesis and thus suggested that more data is needed (BF_10_ = 0.595). The groups did not differ in their scores on the Cognitive-perceptual (Cannabis = 8.813, No Cannabis = 6.731, *p* = 0.13, *r*_*rank*−*biserial*_ = 0.117 [−0.033, 0.262], BF_10_ = 0.234), Interpersonal (Cannabis = 13.125, No Cannabis = 12.900, *p* = 0.82, *r*_*rank*−*biserial*_ = −0.017 [−0.166, 0.133], BF_10_ = 0.143), or total SPQ scales (Cannabis = 24.750, No Cannabis = 20.333, *p* = 0.20, *r*_*rank*−*biserial*_ = 0.099 [−0.051, 0.245], BF_10_ = 0.242). Moreover, the Bayesian analyses suggested that there was moderate evidence for the null hypothesis for these latter three effects (all BF_10_ < 0.3). Similarly, those who currently use cannabis demonstrated significantly higher scores for total ASI (11.000) relative to those who do not (8.375) at a small effect size (*p* = 0.004, *r*_*rank*−*biserial*_ = 0.221 [0.073, 0.359]), but again the BF was insensitive (BF_10_ = 1.934). When analysing the five ASI subscales, the Increased Significance (Cannabis = 3.700, No Cannabis = 2.444, *p* = 0.01, *r*_*rank*−*biserial*_ = 0.202 [0.054, 0.342, BF_10_ = 0.954), Senses Sharpening (Cannabis = 1.955, No Cannabis = 1.000, *p* < 0.001, *r*_*rank*−*biserial*_ = 0.263 [0.117, 0.397], BF_10_ = 3.383), Impending Understanding (Cannabis = 1.900, No Cannabis = 1.316, *p* = 0.02, *r*_*rank*−*biserial*_ = 0.171 [0.021, 0.313], BF_10_ = 0.606), and Heightened Emotionality (Cannabis = 3.033, No Cannabis = 2.311, *p* = 0.03, *r*_*rank*−*biserial*_ = 0.169 [0.019, 0.311], BF_10_ = 0.575) sub-scales were all significantly higher in current cannabis users. However, most of the BF calculations suggested that more data were needed. The Heightened Cognition scale however was non-significant (Cannabis = 1.063, No Cannabis = 0.871, *p* = 0.25, *r*_*rank*−*biserial*_ = 0.085 [−0.066, 0.232], BF_10_ = 0.219), with the BF suggesting moderate evidence to accept the null hypothesis.

### Cannabis Use and KB Performance

**Figure 1:**
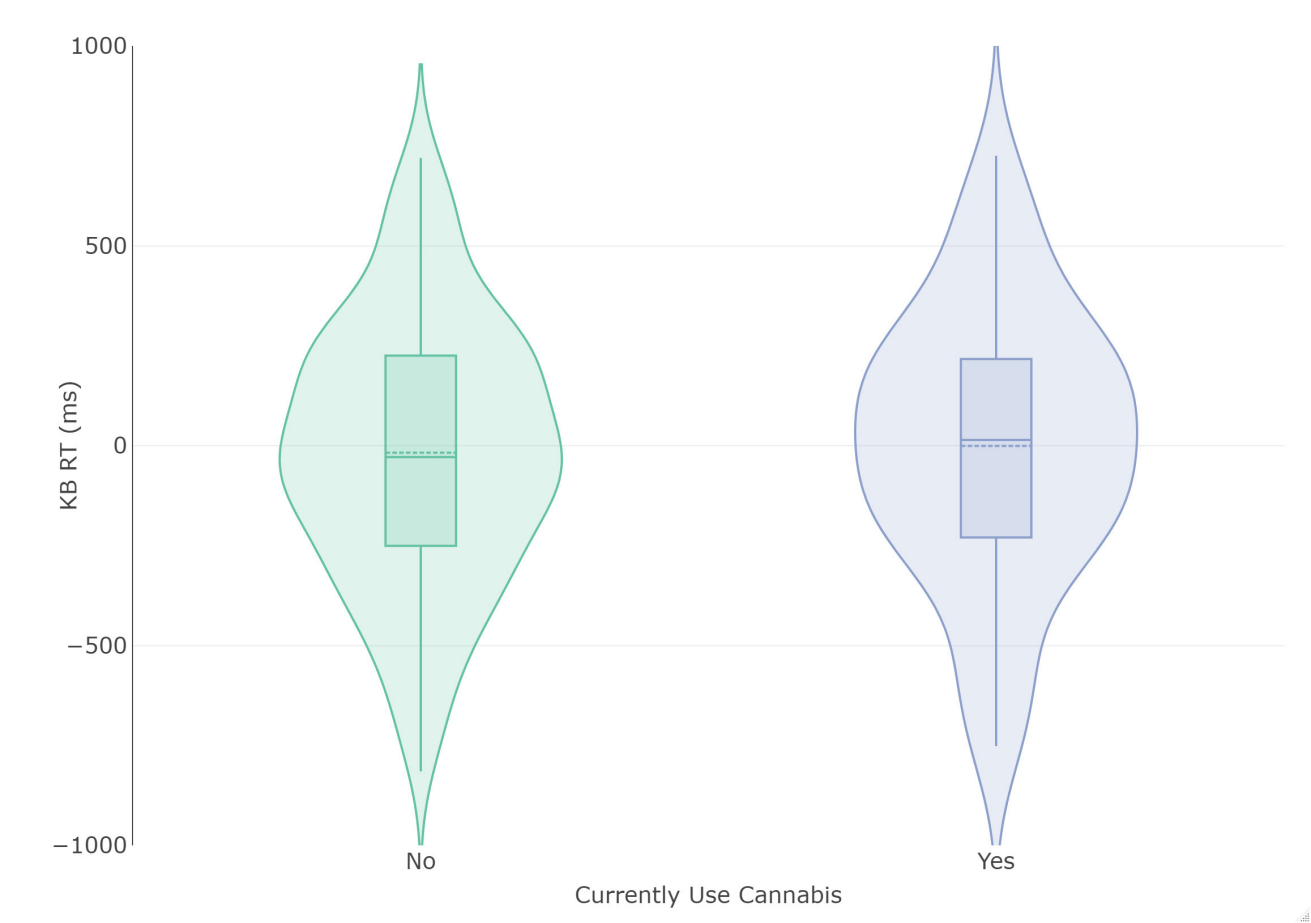
Violin and box plots of KB scores in relation to current use (*n* = 74) and non-use (*n* = 231) of cannabis. The solid line represents the median and dotted line the mean.

Subjects that do not currently use cannabis (*n* = 231) had a lower total KB score (Mean [M]= −17.90 ms, Standard Deviation [SD] = 328.81) than those that do currently smoke cannabis (*n* = 74, M = −0.69, SD = 339.15 ms), but this difference did not attain statistical significance and produced a small effect size with large confidence intervals (mean difference = −17.21 ms, t (303) = −0.389, *p* = 0.70, *d* = −0.052 [−0.31, 0.21]; Figure 1). Homogeneity of variance was not violated (*p* = 0.81). The Bayesian adaptation suggested there was 6.39 –fold more (“moderate”) evidence for the null hypothesis than the alternate hypothesis (Cauchy prior scale = 0.71, BF_10_ = 0.16). As a robustness check, a sequential analysis was performed at different prior settings to assess if these results were stable across participants and priors. This analysis suggested the evidence was insensitive (3 > BF <0.333) until approximately the 120 th participant, after which the evidence for the null increased steadily to moderate strength.

The analysis was repeated for those who stated that they had tried cannabis at least once (but may or may not currently use cannabis i.e., lifetime cannabis use). Those who had never used cannabis (*n* = 125) had a lower mean KB Score (M = −21.61 ms, SD = 315.83 ms) than those that had smoked cannabis (*n* = 179, M = −9.95 ms, SD = 342.02), but this difference did not attain statistical significance and produced a very small effect size with large confidence intervals (mean difference = −11.66, t(302) = −0.302, *p* = 0.76, *d* = −0.035[−0.26, 0.193]). Homogeneity of variance was again not violated (*p* = 0.28). The Bayesian adaptation suggested there was 7.467 more evidence for the null hypothesis (Cauchy prior scale = 0.707, BF_10_ = 0.13). The sequential analysis found evidence for the null increased steadily and was consistent across prior setting.

**Figure 2:**
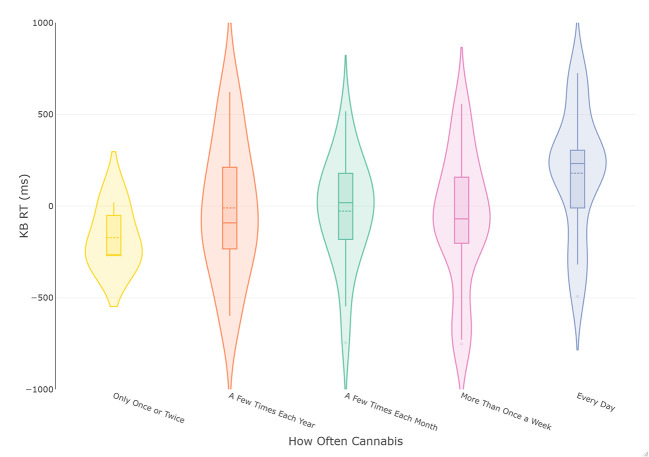
Violin and box plots of KB scores in relation to frequency of cannabis use in current smokers (*n* = 74). The solid line represents the median and dotted line the mean.

The analysis was repeated with cannabis use frequency of the most recent period of cannabis consumption as the grouping variable. This led to the removal of participants reporting they do not currently use cannabis, leaving 74 complete cases. These data were plotted in Figure 2, which appeared to indicate a more consistent increase in KB score with increased cannabis use frequency. The following ANOVA met the assumptions of homogeneity of variance (*p* > 0.51) and normally distributed residuals. The results of the frequentist analysis returned a non-significant effect of cannabis use frequency at a medium effect size [F(4, 68) = 1.45, *p* = 0.23, $\eta_{\text{partial}}^{2}$ = 0.08]. The Bayesian ANOVA found the most probable model was the null model (BF_10_ = 2.74) rather than the proposed model containing cannabis use frequency (BF_10_ = 0.37), with the R^2^ of the model being small (R^2^ = 0.06 [0.007, 0.17]). However, as the BF_10_ was insensitive, more data is needed before conclusions can be drawn.

A follow-up analysis was conducted between current cannabis use and individual KB trial data (trials 1 through 12) by applying a linear mixed-effects model to the data. As we also planned to assess interactions with interpersonal (i.e., negative) schizotypy, two participants needed to be removed who failed to respond to the questionnaire (*n* = 305). To assess whether cannabis use affected trial RT, increasingly complex nested models were compared. At each comparison, a significant χ^2^ difference test suggests the more complex model was a better fit to the data. Akaike Information Criterion (AIC) values are also reported, with lower values indicating a more parsimonious model. Due to the aim of this analysis being model comparison, restricted maximum likelihood (REML) was not used. In all following analyses, inspection of qqplots suggested residuals were approximately normal. The first model contained no fixed effects (independent variables) and only one random effect of participant (random intercept model). This random intercept model was the null model for future comparisons (AIC: 55593), which also allows estimation of the proportion of variance in trial RTs explained by participant's different baseline RTs (i.e., their personal intercepts). The analysis found that the random effect of participant explained 0.337% of the total variance, suggesting little individual differences in baseline RT. Next, this model was compared to a second model also containing trial type as a fixed effect (“main effect”). This second model was a significantly better fit to the data than the null model [χ^2^(11) = 58.2, *p* < 0.001, AIC: 55557] and explained an additional 1.96% of variance (totalling 2.297%). This suggested the extent of KB differed between trials. A further model was proposed that investigated a potential interaction between cannabis use and trial type. This model was not significantly better than the previous model [χ^2^(12) = 13.52, *p* = 0.33, AIC:55568] and explained an additional 0.42% of the variance (total 2.717%). Using the ANOVA function from the base R stats package, the fixed effect of trial type remained significant in this model [F(11, 3093) = 4.78, *p* < 0.001], while both the fixed effect of cannabis use [F(1, 3356) = 0.003, *p* = 0.96] and the interaction of current cannabis use and trial type were non-significant [F(11, 3094) = 1.149, *p* = 0.32]. As it was predicted that interpersonal (negative) schizotypy would predict KB score, we assessed a final model adding interpersonal schizotypy and its interaction with both cannabis use and trial type (three-way interaction). This model was not a significantly better fit to the data relative to the previous model [χ^2^(24) = 21.54, *p* = 0.61, AIC: 55594, R^2^ = 3.310]. Overall, the analysis suggests that RTs differ by trial type but not by cannabis use, interpersonal schizotypy, nor the interaction of these variables.

### Schizotypy, Aberrant Salience and KB Performance

**Figure 3:**
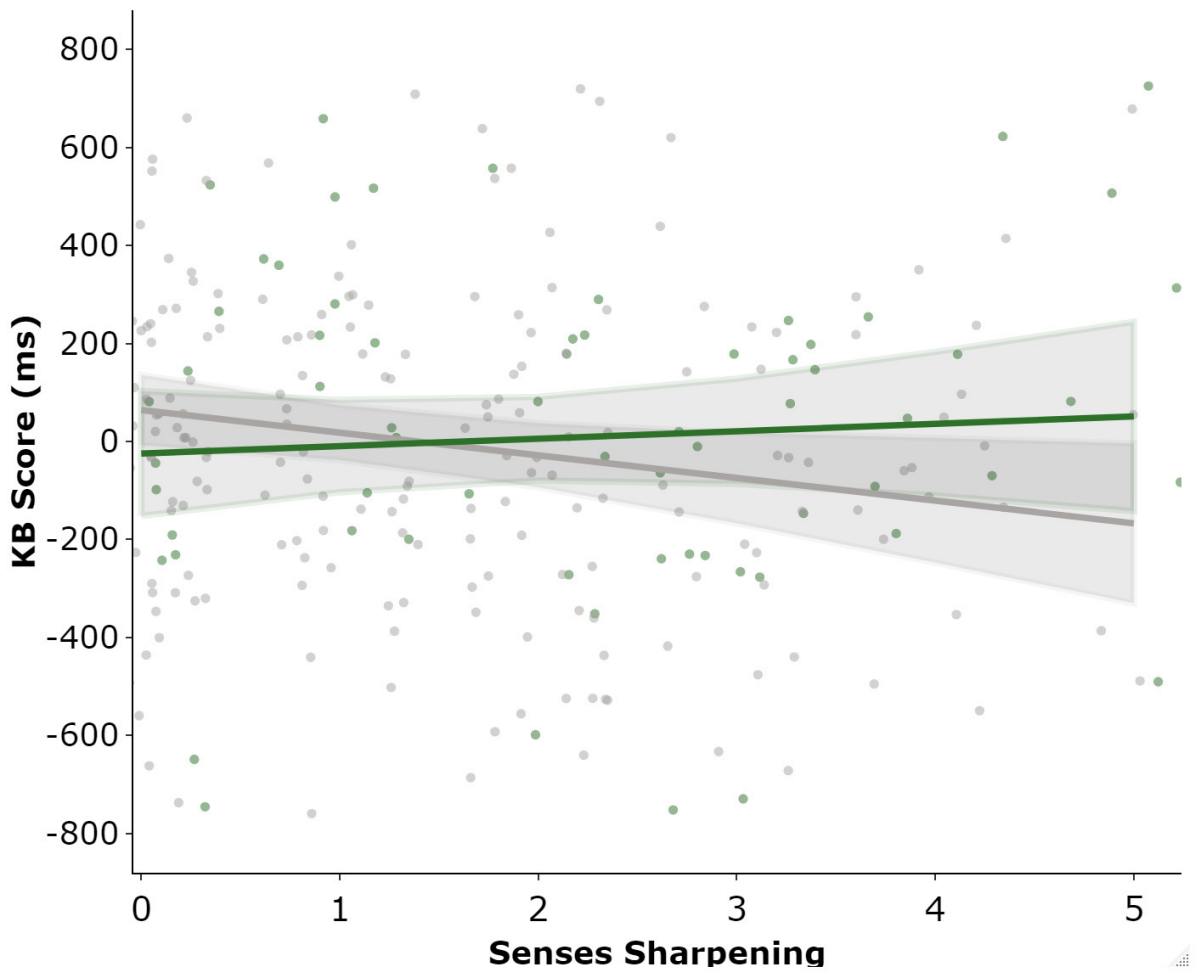
Marginal post estimation of KB as predicted by the ASI “Senses Sharpening” sub-scale. Green line represent current cannabis users and grey line reflects those not currently using cannabis.

Regression analysis was used to examine whether the total and subscale totals of the ASI and subscales of the SPQ were predictors of total KB Score. Consistent with the previous analyses, BFs were also calculated for regression coefficients. Each of the following regression models passed the assumptions of no autocorrelation, homoscedasticity, normal distribution of residuals, lack of outliers/influential points, and no multi-collinearity. The first set of linear regressions entered total ASI (simple linear regression) or its five subscales (multiple regression) as predictors of KB Score. Total ASI score did not predict KB Score (β = 0.037[-0.08, 0.15], *p* = 0.53) and explained little variance (R^2^ = 0.001), with the BF supporting the null (BF_10_ = 0.15). As ASI scores were increased in those that currently use cannabis, we investigated a potential interaction effect between ASI and cannabis use in an additional regression. There was trend level evidence that this model was a better fit to the data [F(2, 298) = 2.533, *p* = 0.08], which likely came from the significant interaction effect between ASI and cannabis use (B = 12.589 [1.505, 23.674], *p* = 0.026). ASI score remained a non-significant predictor (B = −2.220 [-8.202, 3.761], *p* = 0.466) and current cannabis use returned trend (B = −131.524 [−287.637, 24.590], *p* = 0.098). To discern whether this effect was driven by a total ASI or an individual subscale, the analysis was repeated using the ASI subscales as predictors. As calculating the interaction between all ASI subscales and cannabis use status would be unnecessarily complex, a binary logistic regression with cannabis use status as the outcome variable and the ASI subscales as predictors was implemented. This was used to determine whether the association between cannabis use and ASI subscales was general (1 > scale) or specific (only one scale) and thus which interactions to calculate. The binary logistic regression suggested that only Senses Sharpening was a significant predictor of cannabis use status (OR = 1.413, *p* = 0.008), whereas the remaining three scales were not (all *p* > 0.279). Consequently, only the interaction effect between Senses Sharpening and cannabis use was included. The regression analysis (Table 2) found that the only ASI subscale that predicted KB score was Senses Sharpening (B = −46.923 [-85.9, −7.91], *p* = 0.019) and all other subscales returned non-significant (all *p* > 0.20). Current cannabis use did not significantly predict KB score (*p* = 0.20), however, there was a significant interaction effect between cannabis use and Senses Sharpening (B = −61.146 [5.388, 116.9], *p* = 0.032). To illustrate this interaction effect, post-estimation of marginal effects was conducted. As can be seen from Figure 3, in those who do not currently use cannabis Senses Sharpening predicted decreased KB score, whereas Senses Sharpening appeared unrelated to KB score in people who do currently use cannabis.

**Table 2:** Multiple linear regression predicting KB score from scores for ASI subscale scores Increased Significance (Increased Sig), Senses Sharpening (Senses), Impending Understanding (Understanding), Heightened Emotionality (Emotionality), Heightened Cognition (Cognition), as well as current cannabis use and the interaction between current cannabis use and scores on the Senses Sharpening sub-scale [cannabis (yes) ^*^ senses].

	**KB (ms)**				**95% Conf Int**				
**predictor**	**B**	**SE**	**t**	***p***	**LC**	**HC**	$R_{partial}^{2}$	**VIF**	**β**
Intercept	−17.869	36.102	−0.495	0.621	−88.9	53.2			
Increased Sig	9.033	16.548	0.546	0.586	−23.54	41.6	0.101	3.899	0.062
Senses	−46.260	20.236	−2.286	0.023	−86.1	−6.435	0.000	2.533	−0.209
Understanding	−3.460	20.476	−0.169	0.866	−43.8	36.8	0.010	3.106	−0.017
Emotionality	20.647	16.560	1.247	0.214	−11.9	53.2	0.526	2.552	0.114
Cognition	−3.518	20.693	−0.170	0.865	−44.2	37.2	0.010	2.235	−0.015
Cannabis (Yes)	−88.815	68.939	−1.288	0.199	−224.5	46.9	0.561	2.468	−0.116
Cannabis (Yes) * Senses	61.414	28.422	2.161	0.032	5.477	117.4	1.563	3.209	0.222

*F(7, 294) = 1.367; p = 0.219; R_2_ = 3.15%; R_2_ Adjusted = 0.847%; VIF, Variance Inflation Factor*.

Next, the three SPQ dimension scores were added as predictors of KB score in a separate multiple linear regression (Table 3). The model was significantly greater than the null model of intercept alone (F(3, 299) = 2.861, *p* = 0.04), explaining 2.8% of the total variance (adjusted R^2^ = 1.8%). This effect derived primarily from the disorganised scale, in which for every additional item endorsed on the 16 item scale, participant's KB reaction time increased by 17.645 ms (β = 0.237 [0.077, 0.397], *p* =0.004, BF_10_ = 2.474). However, the BF suggests more data are needed to clarify the robustness of this effect. In terms of partial R^2^ values, the Disorganised dimension also accounted for the vast majority of the model's total R^2^ (${\text{R}}_{\text{partial}}^{2}$ = 2.755%). Of the remaining dimensions, the Cognitive-perceptual (β = −0.076 [−0.244, 0.093], *p* = 0.38, BF_10_ = 0.358) and Interpersonal dimensions (β = −0.096 [−0.259, 0.068], *p* = 0.25, BF_10_ = 0.447) both returned non-significant and insensitive. As disorganised schizotypy was increased in current cannabis users, a further model was tested including an interaction effect between current cannabis use and disorganised schizotypy. However, this model was not a significantly better fit to the data (F(2, 297) = 0.416, *p* = 0.66), the interaction effect returned non-significant (B = 8.422 [−10.7, 27.6], *p* = 0.39), and the model explained minimal additional variance (R^2^ = 3.062%). This suggested the model in Table 3 was the most parsimonious.

**Table 3:** Multiple linear regression predicting KB score from scores for SPQ scores for dimensions Cognitive-Perceptual (Cog Perceptual), Interpersonal (Int Personal), and Disorganised.

	**KB (ms)**								**95% Conf Int**	
**predictor**	**B**	**SE**	**t**	***p***	**BF_**10**_**	$R_{partial}^{2}$	**VIF**	**β**	**LC**	**HC**
Intercept	−25.258	36.274	−0.696	0.487						
Cog Perceptual	−3.464	3.916	−0.885	0.377	0.358	0.261%	2.245	−0.076	−0.244	0.093
Int Personal	−3.812	3.319	−1.149	0.252	0.447	0.439%	2.128	−0.096	−0.259	0.068
Disorganised	17.645	6.063	2.911	0.004	2.474	2.755%	2.038	0.237	0.077	0.397

*F(3, 299) = 2.861; p = 0.04; R^2^ = 2.8%; $R_{partial}^{2}$ = 1.8%; VIF, variance inflation factor; priors are full Cauchy (location = 0, scale = 0.354)*.

The final analysis added the SPQ scales, ASI scales, and KB trial scores into a Bayesian correlation matrix using two-tailed Kendall's Tau. Correlations were used to reduce the complexity of the large number of comparisons, Kendall's Tau was chosen due to the positive skew of the SPQ and ASI scales, and the Bayesian approach was applied as BFs do not need correction for multiple comparisons (60). A beta distribution prior was used where all correlation coefficient values were equally likely (a stretched prior width of 1 and robustness check at 0.5). As can be seen from the correlation matrix in Supplementary Table 2A, total SPQ (*r*_*Kendall*_ = 0.032, BF_10_ = 0.106) and both its Cognitive-perceptual (*r*_*Kendall*_ = 0.016, BF_10_ = 0.082) and Interpersonal (*r*_*Kendall*_ = 0.007, BF_10_ = 0.076) dimensions remained unrelated to KB scores, with moderate to strong levels of evidence. The previous significant prediction of KB Score by Disorganised SPQ scores was insensitive in the current correlations (*r*_*Kendall*_ = 0.099, BF_10_ = 2.064). When assessing individual trial correlations, the source of this effect was unclear, although the largest (but insensitive) correlation belonged to its relationship with Trial 6 (*r*_*Kendall*_ = 0.106, BF_10_ = 1.974). For the SPQ subscales, Odd speech was associated with both Trial 6 (*r*_*Kendall*_ = 0.139, BF_10_ = 31.084) and overall KB Score (*r*_*Kendall*_ = 0.119, BF_10_ = 8.959). These associations with Odd Speech likely explain the link between the Disorganised dimension and KB Score in the previous regressions, as well as strengthen the idea that the link between Disorganised and KB Score is through Trial 6. Total ASI levels were largely unrelated to trial scores, with the null hypothesis supported for 11 of the 13 variables. The ASI subscales followed a similar pattern, with 82% of BFs accepting the null and 16.9% being insensitive, although one correlation between Heightened Emotion and Trial 8 had moderate evidence (*r*_*Kendall*_ = 0.115, BF_10_ = 4.339).

Finally, we repeated this correlation matrix in current cannabis users (Supplementary Table 2B). This was conducted to assess whether the previous associations in Supplementary Table 2A remain stable between those who do and do not use cannabis. Briefly, the correlation between disorganised schizotypy and KB score remained insensitive, but descriptively increased in effect size (*r*_*Kendall*_ = 0.150, BF_10_ = 0.874). The association between Odd Speech and Trial 6 remained a similar effect size but became insensitive, which most likely reflects the reduction in sample size (*n* = 74) rather than moderation. A new association was reported between the Heightened Cognition subscale of the ASI and KB score (*r*_*Kendall*_ = 0.210, BF_10_ = 4.605), which primarily came from the association to Trial 6 (*r*_*Kendall*_ = 0.297, BF_10_ = 66.703). Although the remaining subscale were not found to be related to overall KB score (*r*_*Kendall*_ = 0.080–0.152, BF_10_ = 0.205–0.904), associations were found between Trial 6 and both Increased Significance (*r*_*Kendall*_ = 0.277, BF_10_ = 29.806) and Senses Sharpening (*r*_*Kendall*_ = 0.231, BF_10_ = 6.056), with Impending Understanding (*r*_*Kendall*_ = 0.168, BF_10_ = 1.115) and Heightened Emotion (*r*_*Kendall*_ = 0.181, BF_10_ = 1.506) returning insensitive. These correlations corroborate the both differences in SPQ and ASI scores between cannabis users and non-users (replication analyses section) and the significant interaction effect reported between current cannabis use and ASI when predicting KB score.

## Discussion

Based on the literature, we expected regular cannabis users to exhibit reduced KB performance (i.e., increased learning to the added compound stimulus, CS2) relative to non-using participants. We further predicted that selected schizotypal traits and aberrant salience status (from the ASI) would be associated with total and/or individual trials scores in the KB task. Consistent with previously published data (15), current cannabis use (frequent use during preceding year) was associated with higher aberrant salience total and sub-scale scores, along with higher Disorganised scale scores on the SPQ. However, the present study demonstrated that neither current nor lifetime cannabis use was associated with any significant changes in KB performance. KB performance was modestly predicted by scores on the disorganised SPQ dimension. It was also demonstrated that among current cannabis non-users, higher scores on the ASI “Senses Sharpening” [reflecting greater perceptual salience, conceptually related to sensory overload reported by patients with psychosis; (54)] predicted decreased KB score, in a manner not observed in people who do currently use cannabis.

A review of studies measuring (directly or indirectly) at least one of three categories of salience (attentional, affective or motivational salience) has reported limited behavioural evidence for a long-term effect of cannabis use on each salience type (62). With respect to attentional salience, long-term cannabis users show unaffected stroop task performance relative to non-user controls (63–65). Other studies have shown deficits in performance of a task measuring attentional inhibition [negative priming; (32)] or attention-modulated deficits in prepulse inhibition (66) in relatively small samples of regular cannabis users (32). Accounts of impaired salience processing in patients with psychosis or susceptible individuals have incorporated deficits across various information-gating and early attentional processing phenomena (6). Long-term cannabis use has been suggested to lead to increased detection and significance assigned to stimuli that should have been filtered out (67). Via inhibition of glutamate release onto gamma-aminobutyric acid (GABA) neurons in the VTA and NAc, cannabis exposure is indirectly associated with sub-cortical dopaminergic dysregulation, and related salience-based disturbances in psychosis (6). In a report by Jager et al. (68), behavioural and neuroimaging measurements were collected during a visuo-auditory selective attention task (and other cognitive measures) in frequent cannabis users. They reported no effect of regular cannabis use on selective attention task performance but significant alteration in brain activity in selected cortical areas during cognitive performance. These findings may lead to speculation that cannabis effects on attentional salience processes may be best interrogated by neuroimaging or behavioural methods, or perhaps a combination of both. Overall, variable results may also reflect individual differences in cannabis use history (amount and strength of cannabis, duration and frequency of use, etc.), as well as lack of clarity regarding the extent to which these various behavioural measures (and their underlying attentional processes) overlap.

With respect to motivational salience, recent neuroimaging studies have documented acute and chronic cannabinoid effects on “liking” (disposition towards rewarding stimuli), “wanting” (reward seeking behaviour), and reward-related learning behaviour, as well as related activation in selected brain areas (62). Jager et al. (69) found that adolescent cannabis users showed higher levels of brain activity in the caudate and putamen relative to controls during anticipation to both reward and neutral stimuli in a monetary incentive delay task; striatal hyperactivity was reflected in a reduced ability among cannabis users to disengage this circuitry in the absence of the opportunity to gain a reward. In contrast, Filbey et al. (70) reported that cannabis users showed enhanced reward responses towards cannabis-related cues but no evidence of reduced sensitivity towards non-drug rewards. Studies in animal models have demonstrated that prolonged cannabis exposure during young adulthood can produce deficits in motivational function that do not persist into adulthood [e.g., (71)]. These findings are consistent with reports of intact motivation in long-term cannabis users (72). Lawn et al. (73) demonstrated subtle deficits in associative learning in chronic cannabis users, but the present study failed to show similar effects.

In contrast with previous findings, KB performance was not associated with either positive or negative schizotypal traits. Data from patients with schizophrenia and schizotypal participants have demonstrated that a non-paranoid and negative symptom profile is associated with reduction in KB (36, 48), while others have shown an association between KB performance and frequency or level of distress associated with schizotypal delusion-like beliefs (74, 75), as well as cognitive disorganisation (36). In the present study, higher scores on the Disorganised scale of the SPQ were modestly associated with higher KB scores and this effect appeared to be largely a trial-specific effect (i.e., Trial 6). These results are in general agreement with studies that have failed to show any deficits in KB in high schizotypal individuals (49). The study by Humpston and colleagues used a blocking task and an analytic approach that had previously been used to link KB with the negative dimension of schizotypy (48), but they failed to replicate this association. In the present study we employed a modified version of the KB paradigm of Oades et al. (46), which has been used to confirm the schizotypy-KB association (36). Lack of agreement across studies likely reflects several factors. Firstly, different measures of schizotypy have been employed; we measured schizotypy using the SPQ, whereas several of the other studies demonstrating a link between schizotypy and KB employed the Oxford-Liverpool Inventory of Feelings and Experiences (O-LIFE) (36, 48). It has also been proposed that variability may additionally reflect use of different paradigms to measure KB. A series of studies by Jones and colleagues (44, 45) demonstrated inconsistencies with respect to the association between KB and schizotypy that depended upon use of a within- or between-participants design using their task. Additionally, differences across studies may also relate to heterogeneous study populations and the complex challenge (an area for future study) of parsing the influence on associative learning of nicotine use (which may affect dopamine-dependent prediction error signalling), alcohol consumption and other recreational drug use (48).

Here we observed that none of the KB measures were related to variation in ASI total or sub-scale scores. The exception was the “Senses Sharpening” sub-scale score, which negatively predicted KB score in only those who do not currently use cannabis. The absence of a relationship between the ASI total or other sub-scale scores and KB is congruent with a pattern of inconsistent results concerning the relationship between different measures of aberrant salience (76). Previous studies have shown that higher ASI scores are associated with psychotic-like and disorganised symptoms, suspiciousness and social impairment in daily life (77), as well as psychosis-relevant deficits in effort-based decision making (76). However, ASI scores do not correlate with performance in the salience attribution test (SAT), another task-based measure of aberrant salience processing that is influenced by schizotypy (9, 78). In our study, decreased blocking was associated with self-report of aberrant perceptual salience experiences in cannabis non-users only. These findings are consistent with the view that aberrant salience is a multifaceted concept involving a number of dissociable processes (e.g., early attentional, cognitive, affective), which may be differentially disrupted in susceptible populations including frequent cannabis users (62). There is a growing body of literature documenting bidirectional effects of nonacute cannabinoid exposure on attentional and reward processing mechanisms central to KB performance (79). Further research is required to elaborate on interaction between long-term cannabis exposure and other psychosis-linked risk factors on diverse behavioural measures of salience processing, as well as self-reported aberrant salience experiences.

Using a remote testing platform such as Testable Minds enables easier recruitment of a large and more diverse participant sample. However, particularly as it relates to the KB task elements, it introduces a degree of inter-individual variability due to differences in the test setting. The online delivery format and minor adaptations to the task format may have also contributed to failure to replicate previous results examining the relationship of KB performance with schizotypal traits [e.g., (36)]. The overall KB scores in this study were lower than previously reported using supervised laboratory assessments [e.g. (36)]. This lower baseline may have affected the ability to detect any changes. One advantage of online assessment in larger groups is a gain in experimental power. Prior studies suggesting associations between schizotypy and blocking were largely underpowered with the exception of Humpston et al. (49), who reported no correlation between schizotypy and KB using a different blocking task. That study did not use SPQ items related to cognitive disorganisation as measured here. The present study largely agrees with these findings but did identify a weak positive relationship with the Disorganisation dimension.

To conclude, we provide the first evidence for absence of any effect of cannabis use on KB performance in a non-clinical study population. KB performance was also largely unrelated to variation in schizotypy (via SPQ) and aberrant salience (ASI). It has been suggested that psychotic symptoms (e.g., delusions, hallucinations) are related to a generalised inability to attribute salience or associability appropriately and that measurement of prediction error abnormalities (e.g., disrupted blocking) arising from psychotogenic factors may provide a means of accessing this association. Although these null findings need to be interpreted in the context of aforementioned differences with other studies that have examined KB performance in a psychosis research context, the present study furthers our understanding of the relationship between cannabis use history and attentional dysfunction associated with psychosis.

## Data Availability Statement

The raw data supporting the conclusions of this article will be made available by the authors, without undue reservation.

## Ethics Statement

The studies involving human participants were reviewed and approved by Social Research Ethics Committee of University College Cork. Written informed consent for participation was not required for this study in accordance with the national legislation and the institutional requirements.

## Author Contributions

CO'T, PM, and SC were involved in the conception and design of the study. AB, CD, CO'N, CO'T, JW, and PM participated in data collection and interpretation. CD completed the statistical analysis. CD, CO'T, JW, and PM participated in the drafting and critical revision of this article. AB, CO'N, and SC were involved in the critical revision of the manuscript. All authors agreed to be cited as co-authors, accepting the order of authorship, and approved the final version of manuscript and the manuscript submission to Frontiers in Psychiatry.

## Conflict of Interest

The authors declare that the research was conducted in the absence of any commercial or financial relationships that could be construed as a potential conflict of interest.

## Publisher's Note

All claims expressed in this article are solely those of the authors and do not necessarily represent those of their affiliated organizations, or those of the publisher, the editors and the reviewers. Any product that may be evaluated in this article, or claim that may be made by its manufacturer, is not guaranteed or endorsed by the publisher.
