# Supplementary material for: Cannabis Use, Schizotypy and Kamin Blocking Performance
Source: Front Psychiatry. 2021 Nov 23;12:633476. doi: 10.3389/fpsyt.2021.633476 (PMC8649723; doi:10.3389/fpsyt.2021.633476)
Supplement: Supplementary file 1 [file Table_1.DOCX]

**Supplementary Table 1** Further analysis of cannabis and other drug use characteristics

| ***Characteristic*** | *n* | ***%*** |
| --- | --- | --- |
| **Form of cannabis used**  Hash (cannabis resin/solid)  Imported herbal cannabis  Home-grown skunk  Super-skunk  Other | 84  36  29  4  15 | (27.4%)  (11.7%)  (9.4%)  (1.3%)  (4.9%) |
| **Other drugs (legal and illegal) used recreationally currently or previously** |  |  |
| Alcohol | 174 | (56.7%) |
| Nicotine | 97 | (31.6%) |
| Cocaine | 41 | (13.4%) |
| MDMA | 38 | (12.4%) |
| Lysergic acid | 19 | (6.2%) |
| Methamphetamine | 7 | (2.2%) |
| Ketamine | 7 | (2.2%) |
|  |  |  |

*Figures presented are number (%) unless stated otherwise.*
