# Supplementary material for: Cannabis Use, Schizotypy and Kamin Blocking Performance
Source: Front Psychiatry. 2021 Nov 23;12:633476. doi: 10.3389/fpsyt.2021.633476 (PMC8649723; doi:10.3389/fpsyt.2021.633476)
Supplement: Supplementary file 2 [file Table_2.DOCX]

**Supplementary Table 2** Bayesian correlation matrix of the SPQ total score, factor scores, and subscales, with both ASI score and KB RTs

|  | | | | | | | | | | | | | | | | |  |  |  |  |  |
| --- | --- | --- | --- | --- | --- | --- | --- | --- | --- | --- | --- | --- | --- | --- | --- | --- | --- | --- | --- | --- | --- |
|  |  | **SPQ** | | | | | | | | | | | | |  | **ASI** | | | | | |
|  |  | **Total** | **Cognitive**  **Perceptual** | **Inter-**  **personal** | **Dis-**  **organised** | **Ideas**  **Of**  **Reference** | **Excessive**  **Social**  **Anxiety** | **Odd**  **Beliefs** | **Unusual**  **PE** | **Odd**  **Or**  **eccentric** | **No**  **Close**  **friends** | **Odd**  **speech** | **Constricted**  **affect** | **Suspi-**  **ciousness** |  | **Total** | **IS** | **SS** | **IU** | **HE** | **HC** |
| *ASI* |  | 0.480D | 0.562D | 0.324D | 0.419D | 0.464D | 0.227D | 0.406D | 0.545D | 0.363D | 0.239D | 0.403D | 0.250D | 0.451D |  | - |  |  |  |  |  |
| *ASI_IS* |  | 0.438D | 0.510D | 0.290D | 0.401D | 0.453D | 0.209D | 0.349D | 0.506D | 0.351D | 0.210D | 0.381D | 0.214D | 0.415D |  | 0.813D | - |  |  |  |  |
| *ASI_SS* |  | 0.320D | 0.413D | 0.192D | 0.288D | 0.305D | 0.091I | 0.358D | 0.458D | 0.263D | 0.148D | 0.274D | 0.144*** | 0.323D |  | 0.634D | 0.539D | - |  |  |  |
| *ASI_IU* |  | 0.397D | 0.490D | 0.270D | 0.348D | 0.418D | 0.163D | 0.385D | 0.458D | 0.315D | 0.204D | 0.324D | 0.215D | 0.405D |  | 0.739D | 0.683D | 0.536D | - |  |  |
| *ASI_HE* |  | 0.483D | 0.504D | 0.361D | 0.441D | 0.430D | 0.287D | 0.340D | 0.474D | 0.361D | 0.261D | 0.447D | 0.296D | 0.446D |  | 0.717D | 0.600D | 0.422D | 0.53D | - |  |
| *ASI_HC* |  | 0.441D | 0.496D | 0.321D | 0.374D | 0.410D | 0.264D | 0.365D | 0.507D | 0.326D | 0.227D | 0.370D | 0.250D | 0.392D |  | 0.646D | 0.541D | 0.464D | 0.468D | 0.561D | - |
| *KB Score* |  | 0.032N | 0.016N | 0.007N | 0.099I | 0.031N | 0.032N | 0.001N | 0.024N | 0.044N | -0.018N | 0.119* | 0.029N | 0.009N |  | 0.019N | 0.023N | -0.045N | 0.016N | 0.051N | 0.028N |
| *Trial1* |  | 0.034N | 0.016N | 0.025N | 0.034N | 0.069I | 0.037N | 0.045N | -0.035N | 0.012N | 0.020N | 0.049N | 0.045N | 0.001N |  | -0.019N | 0.021N | -0.063N | 0.028N | -0.033N | -0.011N |
| *Trial2* |  | -0.053N | -0.047N | -0.037N | -0.056N | -0.014N | -0.022N | -0.024N | -0.105I | -0.065N | -0.049N | -0.061N | -0.036N | -0.019N |  | -0.094I | -0.095I | -0.102I | -0.082I | -0.070I | -0.094I |
| *Trial3* |  | 0.030N | 0.020N | 0.044N | 0.018N | 0.028N | 0.081I | -0.041N | 0.034N | 0.017N | 0.013N | 0.017N | 0.007N | 0.036N |  | -0.007N | 0.005N | -0.030N | -0.019N | -0.012N | -0.014N |
| *Trial4* |  | -0.033N | -0.029N | -0.032N | -0.023N | -0.044N | -0.064N | -0.010N | -0.017N | -0.035N | 0.001N | -0.026N | -0.017N | -0.008N |  | -0.007N | 0.010N | -0.021N | 0.041N | -0.016N | -0.058N |
| *Trial5* |  | -0.001N | 0.008N | -0.020N | 0.037N | 0.032N | 0.004N | 0.005N | -0.003N | 0.020N | -0.054N | 0.040N | -0.021N | 0.002N |  | 0.007N | 0.033N | -0.015N | 0.002N | -0.005N | -0.012N |
| *Trial6* |  | 0.071I | 0.057N | 0.047N | 0.102I | 0.055N | 0.047N | 0.023N | 0.089I | 0.052N | 0.043N | 0.139*** | 0.040N | 0.046N |  | 0.070I | 0.090I | 0.041N | 0.021N | 0.077I | 0.079I |
| *Trial7* |  | 0.041N | 0.019N | 0.043N | 0.081I | -0.004N | 0.056N | -0.004N | -0.001N | 0.067I | 0.008N | 0.081I | 0.049N | 0.045N |  | 0.047N | 0.029N | 0.025N | 0.063N | 0.064N | 0.051N |
| *Trial8* |  | 0.046N | 0.058N | 0.033N | 0.072I | 0.009N | 0.019N | 0.024N | 0.049N | 0.055N | -0.024N | 0.066I | 0.062N | 0.073I |  | 0.047N | 0.014N | -0.010N | 0.034N | 0.115* | 0.032N |
| *Trial9* |  | 0.008N | 0.007N | 0.017N | -0.005N | 0.044N | 0.011N | -0.055N | -0.017N | -0.006N | 0.027N | 0.008N | 0.014N | -0.002N |  | -0.001N | -0.035N | 0.029N | -0.023N | 0.028N | 0.034N |
| *Trial10* |  | -0.020N | -0.005N | -0.029N | 0.007N | 0.003N | -0.043N | 0.014N | -0.023N | 0.003N | -0.014N | -0.009N | -0.022N | 0.013N |  | -0.026N | -0.038N | -0.051N | 0.002N | -0.029N | -0.017N |
| *Trial11* |  | 0.055N | 0.059N | 0.044N | 0.057N | 0.035N | 0.029N | 0.031N | 0.094I | 0.034N | 0.063N | 0.069I | 0.048N | 0.036N |  | 0.062N | 0.073I | 0.016N | 0.072I | 0.028N | 0.076I |
| *Trial12* |  | -0.007N | -0.014N | 0.017N | -0.001N | -0.012N | 0.021N | 0.027N | -0.052N | 0.000N | 0.012N | -0.006N | 0.029N | 0.021N |  | -0.022N | -0.020N | -0.029N | -0.016N | -0.004N | -0.001N |
|  | ***Note***: ^N^ = supports the Null, ^I^ = insensitive, * BF₁₀ > 3, ** BF₁₀ > 10, *** BF₁₀ > 30, ^D^ = >100 for ‘decisive’. Stretched beta prior width of 1. ASI sub-scales: increased significance (IS), sense sharpening (SS), impending understanding (IU), heightened emotion (HE), heightened cognition (HC) | | | | | | | | | | | | | | | | | | | | |
