# Supplementary material for: Cannabis Use, Schizotypy and Kamin Blocking Performance
Source: Front Psychiatry. 2021 Nov 23;12:633476. doi: 10.3389/fpsyt.2021.633476 (PMC8649723; doi:10.3389/fpsyt.2021.633476)
Supplement: Supplementary file 3 [file Table_3.docx]

**Supplementary Table 3** Bayesian correlation matrix of the SPQ total score, factor scores, and subscales, with both ASI score and KB RTs

|  | | | | | | | | | | | | | | | | |  |  |  |  |  |
| --- | --- | --- | --- | --- | --- | --- | --- | --- | --- | --- | --- | --- | --- | --- | --- | --- | --- | --- | --- | --- | --- |
|  |  | **SPQ** | | | | | | | | | | | | |  | **ASI** | | | | | |
|  |  | **Total** | **Cognitive**  **Perceptual** | **Inter-**  **personal** | **Dis-**  **organised** | **Ideas**  **Of**  **Reference** | **Excessive**  **Social**  **Anxiety** | **Odd**  **Beliefs** | **Unusual**  **PE** | **Odd**  **Or**  **eccentric** | **No**  **Close**  **friends** | **Odd**  **speech** | **Constricted**  **affect** | **Suspi-**  **ciousness** |  | **Total** | **IS** | **SS** | **IU** | **HE** | **HC** |
| *ASI* |  | 0.545D | 0.645D | 0.331D | 0.438D | 0.518D | 0.215* | 0.481D | 0.657D | 0.341D | 0.288D | 0.438D | 0.203* | 0.441D |  | - |  |  |  |  |  |
| *ASI_IS* |  | 0.482D | 0.560D | 0.301D | 0.387D | 0.444D | 0.216* | 0.396D | 0.617D | 0.286*** | 0.272*** | 0.401D | 0.182I | 0.393D |  | 0.800D | - |  |  |  |  |
| *ASI_SS* |  | 0.400D | 0.511D | 0.182I | 0.378D | 0.382D | 0.074N | 0.471D | 0.561D | 0.338D | 0.218* | 0.341D | 0.075N | 0.321D |  | 0.702D | 0.594D | - |  |  |  |
| *ASI_IU* |  | 0.464D | 0.580D | 0.304D | 0.362D | 0.509D | 0.100I | 0.417D | 0.469D | 0.336D | 0.289D | 0.328D | 0.254** | 0.453D |  | 0.678D | 0.587D | 0.562D | - |  |  |
| *ASI_HE* |  | 0.528D | 0.573D | 0.364D | 0.411D | 0.456D | 0.303D | 0.360D | 0.576D | 0.301D | 0.252** | 0.454D | 0.210* | 0.439D |  | 0.678D | 0.578D | 0.435D | 0.446D | - |  |
| *ASI_HC* |  | 0.515D | 0.538D | 0.351D | 0.425D | 0.462D | 0.320D | 0.405D | 0.559D | 0.289D | 0.249** | 0.475D | 0.247** | 0.387D |  | 0.717D | 0.632D | 0.545D | 0.486D | 0.613D | - |
| *KB Score* |  | 0.099I | 0.093N | 0.038N | 0.150I | 0.084N | 0.075N | 0.054N | 0.137I | 0.100I | -0.037N | 0.144I | 0.052N | 0.116I |  | 0.140I | 0.152I | 0.109I | 0.080N | 0.138I | 0.210* |
| *Trial1* |  | 0.139I | 0.102I | 0.146I | 0.099I | 0.106I | 0.074N | 0.080N | 0.035N | 0.041N | 0.125I | 0.122I | 0.195I | 0.144I |  | 0.045N | 0.078N | -0.024N | 0.039N | 0.028N | 0.145I |
| *Trial2* |  | -0.132I | -0.062N | -0.109I | -0.143I | 0.012N | -0.037N | -0.045N | -0.102I | -0.141I | -0.113I | -0.135I | -0.132I | -0.079N |  | -0.108I | -0.116I | -0.144I | -0.043N | -0.094N | -0.132I |
| *Trial3* |  | 0.134I | 0.105I | 0.149I | 0.086N | 0.118I | 0.246** | 0.014N | 0.154I | 0.050N | 0.082N | 0.097I | 0.065N | 0.056N |  | 0.104I | 0.131I | 0.109I | 0.030N | 0.088N | 0.113I |
| *Trial4* |  | 0.010N | -0.027N | -0.029N | 0.057N | -0.043N | -0.089N | -0.047N | 0.031N | 0.088N | 0.063N | 0.001N | 0.014N | -0.074N |  | 0.039N | 0.036N | 0.133I | 0.106I | -0.09N | -0.061N |
| *Trial5* |  | -0.039N | -0.009N | -0.069N | 0.005N | 0.022N | -0.066N | 0.031N | 0.009N | -0.045N | -0.115I | 0.040N | -0.013N | -0.007N |  | -0.005N | 0.072N | -0.011N | -0.056N | -0.016N | -0.017N |
| *Trial6* |  | 0.148I | 0.212* | 0.059N | 0.145I | 0.173I | 0.076N | 0.260** | 0.229* | 0.116I | 0.011N | 0.146I | 0.049N | 0.107I |  | 0.247** | 0.277** | 0.231* | 0.168I | 0.181I | 0.297*** |
| *Trial7* |  | -0.010N | -0.041N | -0.048N | 0.082N | -0.031N | 0.019N | -0.056N | -0.045N | 0.104I | -0.093N | 0.027N | 0.051N | -0.069N |  | 0.040N | 0.019N | -0.051N | 0.022N | 0.081N | 0.126I |
| *Trial8* |  | 0.164I | 0.190I | 0.161I | 0.105I | 0.160I | 0.107I | 0.186I | 0.129I | 0.020N | 0.003N | 0.163I | 0.130I | 0.251** |  | 0.094N | 0.016N | 0.013N | 0.078N | 0.182I | 0.087N |
| *Trial9* |  | 0.059N | -0.022N | 0.108I | 0.058N | 0.016N | 0.125I | -0.204I | 0.004N | 0.034N | 0.090N | 0.077N | 0.186I | -0.025N |  | -0.035N | -0.061N | 0.006N | -0.016N | 0.014N | -0.009N |
| *Trial10* |  | -0.043N | -0.049N | -0.053N | 0.002N | -0.037N | -0.113I | 0.041N | -0.118I | 0.037N | -0.033N | -0.071N | -0.028N | 0.057N |  | -0.097I | -0.110I | -0.028N | -0.038N | -0.126I | -0.082N |
| *Trial11* |  | 0.052N | 0.117I | 0.018N | -0.016N | 0.076N | -0.038N | 0.171I | 0.129I | -0.027N | 0.091N | 0.006N | 0.015N | 0.071N |  | 0.187I | 0.188I | 0.220* | 0.243** | 0.062N | 0.108I |
| *Trial12* |  | 0.101I | 0.049N | 0.136I | 0.069N | 0.058N | 0.217* | 0.050N | 0.016N | 0.010N | 0.100I | 0.073N | 0.054N | 0.072N |  | 0.052N | 0.034N | 0.110I | 0.019N | 0.019N | 0.111I |
|  | ***Note***: ^N^ = supports the Null, ^I^ = insensitive, * BF₁₀ > 3, ** BF₁₀ > 10, *** BF₁₀ > 30, ^D^ = >100 for ‘decisive’. Stretched beta prior width of 1. ASI sub-scales: increased significance (IS), sense sharpening (SS), impending understanding (IU), heightened emotion (HE), heightened cognition (HC) | | | | | | | | | | | | | | | | | | | | |
